# Caveolin-1 is an aggresome-inducing protein

**DOI:** 10.1038/srep38681

**Published:** 2016-12-08

**Authors:** Ajit Tiwari, Courtney A. Copeland, Bing Han, Caroline A. Hanson, Krishnan Raghunathan, Anne K. Kenworthy

**Affiliations:** 1 Department of Molecular Physiology and Biophysics, Vanderbilt University School of Medicine, Nashville, Tennessee, USA; 2 Department of Cell and Developmental Biology, Vanderbilt University School of Medicine, Nashville, Tennessee, USA; 3 Epithelial Biology Program, Vanderbilt University School of Medicine, Nashville, Tennessee, USA; 4 Chemical and Physical Biology Program, Vanderbilt University, Nashville, Tennessee, USA

## Abstract

Caveolin-1 (Cav1) drives the formation of flask-shaped membrane invaginations known as caveolae that participate in signaling, clathrin-independent endocytosis and mechanotransduction. Overexpression or mutations of Cav1 can lead to its mistrafficking, including its accumulation in a perinuclear compartment previously identified as the Golgi complex. Here, we show that in the case of overexpressed Cav1-GFP, this perinuclear compartment consists of cytoplasmic inclusion bodies generated in response to the accumulation of aggregates of misfolded proteins, known as aggresomes. Aggresomes containing Cav1-GFP are encased within vimentin cages, form in a microtubule-dependent manner, and are enriched in a number of key regulators of protein turnover, including ubiquitin, VCP/p97 and proteasomes. Interestingly, aggresome induction was cell-type dependent and was observed for many but not all Cav1 constructs tested. Furthermore, endogenous Cav1 accumulated in aggresomes formed in response to proteosomal inhibition. Our finding that Cav1 is both an aggresome-inducing and aggresome-localized protein provides new insights into how cells handle and respond to misfolded Cav1. They also raise the possibility that aggresome formation may contribute to some of reported phenotypes associated with overexpressed and/or mutant forms of Cav1.

Caveolin-1 (Cav1) is a major structural protein of flask-shaped invaginations known as caveolae, an abundant feature of the plasma membrane in many cell types^1^. Caveolin-1 and caveolae have been proposed to function as regulators of multiple pathways including endocytic trafficking, signaling, lipid homeostasis, and mechanotransduction^2^,^3^. However, a clear consensus model for how Cav1 and caveolae perform these varied functions has yet to emerge^4^.

Cav1 plays an essential role in the formation of a functional caveolae at the plasma membrane. Caveolar biogenesis begins with the insertion of newly synthesized Cav1 into the endoplasmic reticulum where the protein forms oligomers^1^,^5^,^6^,^7^. Cav1 oligomers are subsequently transported to Golgi complex where they associate with cholesterol and form large detergent insoluble complexes, and are finally delivered to the plasma membrane where accessory proteins such as the cavins are recruited to aid in the formation of stable caveolae structures^5^,^8^,^9^,^10^.

Although wild type Cav1 is typically incorporated in caveolae, several Cav1 mutants have been reported to accumulate within the Golgi complex and this mistrafficking event has been attributed to defective oligomerization of Cav1 mutants^11^,^12^,^13^,^14^. Overexpression of wild type (WT) Cav1-GFP is sufficient to induce a similar phenotype^15^,^16^. Under these conditions the protein appears to be poorly folded, forms irregular aggregates, and is rapidly turned over^15^,^16^. This is in striking contrast to the behavior of overexpressed Cav1-mCherry, which is delivered to the plasma membrane as small oligomers that are ubiquitinated and targeted to endolysosomal compartment for degradation in a process that involves Hrs and Tsg101^17^, as well as VCP and UBXD1^18^. These findings suggest that mutations and overexpression of Cav1 interfere with correct targeting of the protein to caveolae and that the fate of Cav1 is also strongly dependent on tagging strategies.

One mechanism utilized by cells to handle misfolded proteins is aggresome formation. Aggresomes are cytoplasmic inclusion bodies that are generated in response to the accumulation of aggregates of misfolded proteins^19^,^20^. Most but not all aggresome-associated proteins have been shown to be ubiquitinated, and depending on the cell types and associated misfolded proteins, aggresomes may contain a variety of chaperones^21^,^22^,^23^. Aggresome formation is typically accompanied by the formation of a cage-like structure composed of intermediate filaments around the aggresome^19^,^20^,^21^. Proteasomes are also often associated with aggresomes^19^,^20^,^23^,^24^,^25^,^26^,^27^. Aggresomes are typically located in the pericentriolar region of the cells near the microtubule-organizing center (MTOC) and their biogenesis is dependent on the microtubule network and cytoplasmic dynein motors^19^,^21^,^23^,^27^. Based on their location, aggresomes could potentially be mistaken for the Golgi complex, as both compartments are localized around MTOC.

In the current study, we show that overexpression of Cav1-GFP induces aggresome formation. These findings have important implications for our understanding of how cells handle and respond to overexpressed and mutant forms of Cav1.

## Results

### Cav1-GFP accumulates in structures with characteristic features of aggresomes

**Figure 1:**
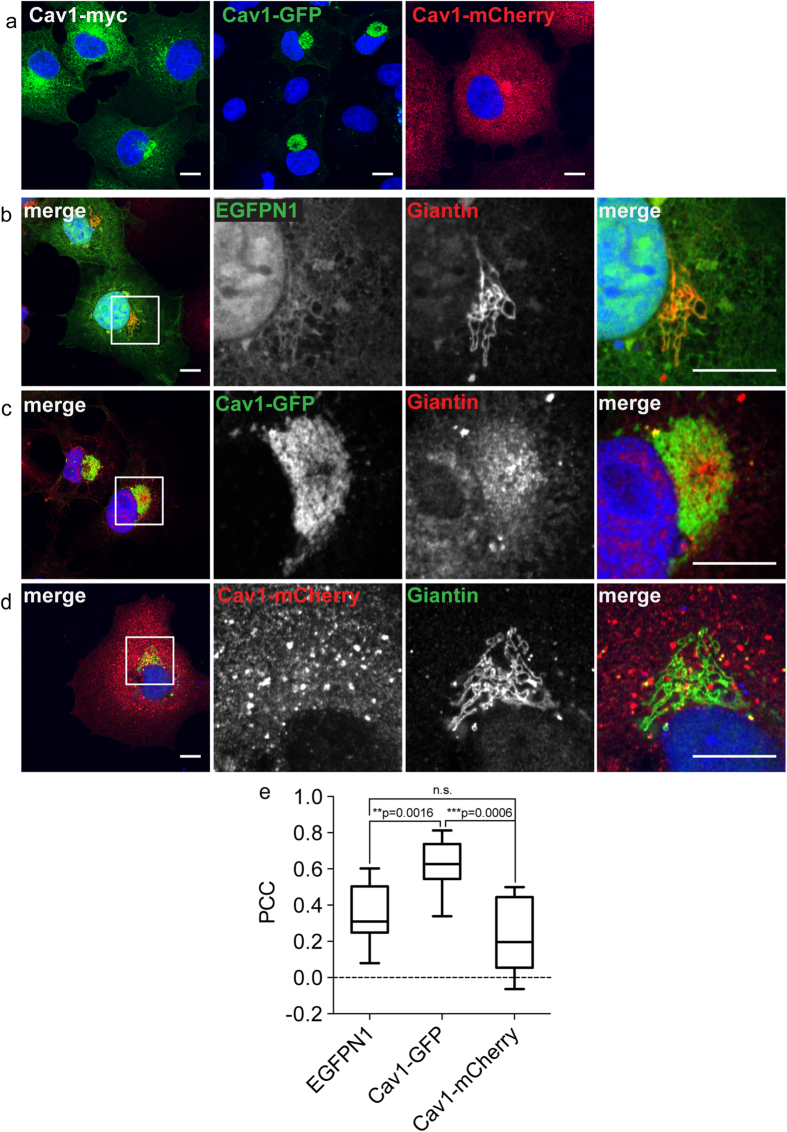
Cav1-GFP accumulates in a perinuclear compartment that partially overlaps with giantin staining. COS-7 cells were transiently transfected with indicated Cav1 constructs were immunostained with indicated antibodies. DRAQ5 was used to label the nucleus (blue). (**a**) Cells expressing Cav1-myc were stained with anti-myc antibody. The fluorescence of Cav1-GFP and Cav1-mCherry was visualized directly. (**b–d**) Cells expressing empty vector (EGFPN1), Cav1-GFP, or Cav1-mCherry were immunostained with an anti-giantin antibody. In the merged images of cells expressing EGFP or Cav1-GFP, GFP fluorescence is shown in green and giantin is shown in red. For the Cav1-mCherry transfected cells, mCherry fluorescence is shown in red and giantin staining is green. White boxes indicate the areas of the zooms. Representative images from at least 2 independent experiments are shown. Scale bars, 10 μm. **(e)** Quantification of colocalization of giantin with the indicated constructs using Pearson’s correlation coefficient (PCC). n.s., not significant, P > 0.05; *P ≤ 0.05; **P ≤ 0.01, ***P ≤ 0.001; Mann-Whitney U-test. n = 8–9 cells per condition.

In a recent study, we showed that overexpressed Cav1-GFP, but not Cav1-mCherry or Cav1-myc extensively accumulates in perinuclear compartments in several cell types^15^,^16^. To study the mechanisms involved in trapping Cav1-GFP intracellularly, we used COS-7 cells as a model. In this cell type, Cav1-GFP is strongly localized to the perinuclear region, whereas Cav1-myc and Cav1-mCherry are typically partially localized to a perinuclear compartment as well as distributed throughout the cell in reticular and/or punctate patterns (Fig. 1a, Supplementary Fig. S1). In contrast, in untransfected cells endogenous Cav1 is found in punctate structures with an appearance typical of caveolae (Supplementary Fig. S1). These findings confirm previous reports that overexpression of Cav1 leads to mislocalization of the protein^17^ and that overexpressed Cav1-GFP in particular tends to accumulate in a perinuclear compartment^15^,^16^.

Mutants of Cav1 have often been reported to accumulate in the Golgi complex^11^,^12^,^13^,^14^. To test whether Cav1-GFP is trapped in the Golgi complex, we asked whether it colocalizes with the Golgi marker giantin. Immunostaining revealed that the Cav1-GFP-positive compartment overlapped with giantin staining (Fig. 1c,e). However, giantin staining appeared to be disorganized in cells expressing Cav1-GFP (Fig. 1c) compared to cells expressing EGFPN1 or Cav1-mCherry (Fig. 1b,d). The disorganization of Golgi structure in cells overexpressing Cav1-GFP was confirmed by super resolution microscopy of giantin labeling (Supplementary Fig. S2). Super resolution images showed that indeed, Golgi structure is disrupted upon overexpressing of Cav1-GFP, but not EGFPN1 or Cav1-mCherry (Supplementary Fig. S2). These results indicate that Cav1-GFP associates with a structure that is in proximity to, but possibly distinct from the Golgi complex, and also suggest that expression of Cav1-GFP disrupts the Golgi complex.

Our previous studies suggested that perinuclear Cav1-GFP is present in a misfolded and aggregated form as evidenced by biochemical fractionation and epitope accessibility studies^15^,^16^. One of the compartments proposed to house aggregated proteins near the perinuclear region is the aggresome^19^,^21^. Aggresome formation is often accompanied by redistribution of the intermediate filament protein vimentin, which forms cages around aggresomes^19^,^21^,^23^. In addition, aggresome formation can lead to disruption of the Golgi complex^21^. We therefore hypothesized that the Cav1-GFP-positive structures might be aggresomes.

**Figure 2:**
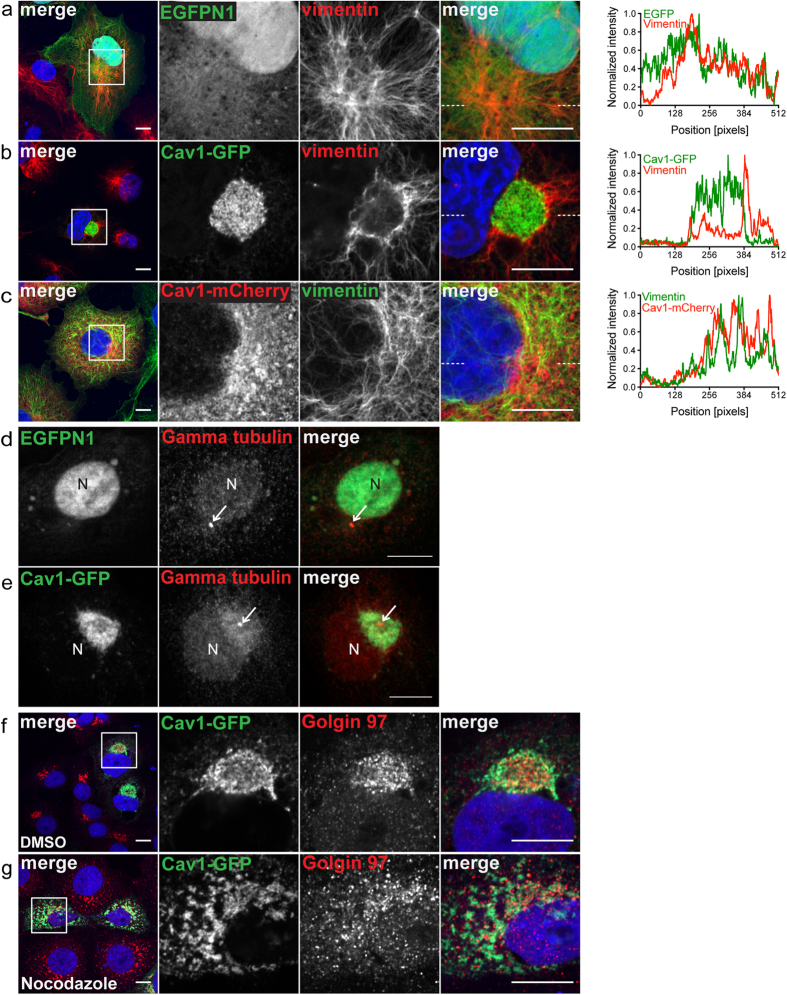
The perinuclear pool of Cav1-GFP is encased within a vimentin cage and is localized around the microtubule-organizing center in a microtubule-dependent manner. COS-7 cells transiently transfected with empty vector (EGFPN1), Cav1-GFP, or Cav1-mCherry were stained with the indicated antibodies. (**a–c**) Distribution of endogenous vimentin in transfected cells. In the merged images of cells expressing EGFP or Cav1-GFP, GFP fluorescence is shown in green and vimentin is shown in red. For the Cav1-mCherry transfected cells, mCherry fluorescence is shown in red and vimentin staining is green. DRAQ5 was used to label the nucleus (blue). White boxes indicate the areas of the zooms. Line scan intensity profiles for a-c are shown on the far right. The position of line scan is indicated by the dotted lines at the edges of the merged image. (**d,e**) Cells overexpressing EGFP or Cav1-GFP (green) were immunostained using an anti-gamma tubulin antibody (red). “N” indicates the position of the nucleus. The position of MTOC is indicated by white arrows. (**f,g**) Cav1-GFP transfected COS-7 cells were treated with (**f**) DMSO or (**g**) nocodazole for 1 h at 37 °C. The cells were then fixed, permeabilized and stained with the Golgi marker golgin 97. Representative images are shown. In the merged images Cav1 staining is shown in green and golgin-97 staining is depicted in red. Nuclei are labeled blue. Representative images from 2 independent experiments are shown. Scale bars, 10 μm.

To test this, we immunostained Cav1-GFP overexpressing cells for endogenous vimentin (Fig. 2). Vimentin reorganized into cage-like structures around perinuclear Cav1-GFP and was excluded from the interior of the Cav1-GFP compartment (Fig. 2b). This phenotype is similar to those observed previously for aggresomes^19^,^21^. In contrast, vimentin cages were not observed in cells expressing EGFP (Fig. 2a) or Cav1-mCherry (Fig. 2c). This was further corroborated by line scan intensity profiles across the perinuclear region (Fig. 2a–c). Furthermore, Cav1-GFP was localized around the MTOC as marked by gamma tubulin staining (Fig. 2e), a typical location of aggresomal structures, and maintenance of this perinuclear localization was dependent on the presence of an intact microtubule network (Fig. 2f,g), another feature of aggresomes^19^,^20^,^21^. Taken together, these results suggest that Cav1-GFP is not simply localized to the Golgi complex under these conditions. Rather, it induces aggresome formation.

### A variety of GFP-tagged Cav1 constructs induce aggresome formation

The finding that Cav1-GFP overexpression induces aggresome formation raised the question of whether this behavior is specific to this particular construct or is a more general property of GFP-tagged forms of Cav1. To address this question we examined the effect of overexpression of several additional Cav1 constructs previously described in the literature^15^,^28^. As summarized in Supplementary Table S1, these constructs contained Cav1 cDNA derived from different species, different forms of GFP, including the monomeric GFP variant mEmerald, and both N-terminally and C-terminally tagged versions of Cav1.

**Figure 3:**
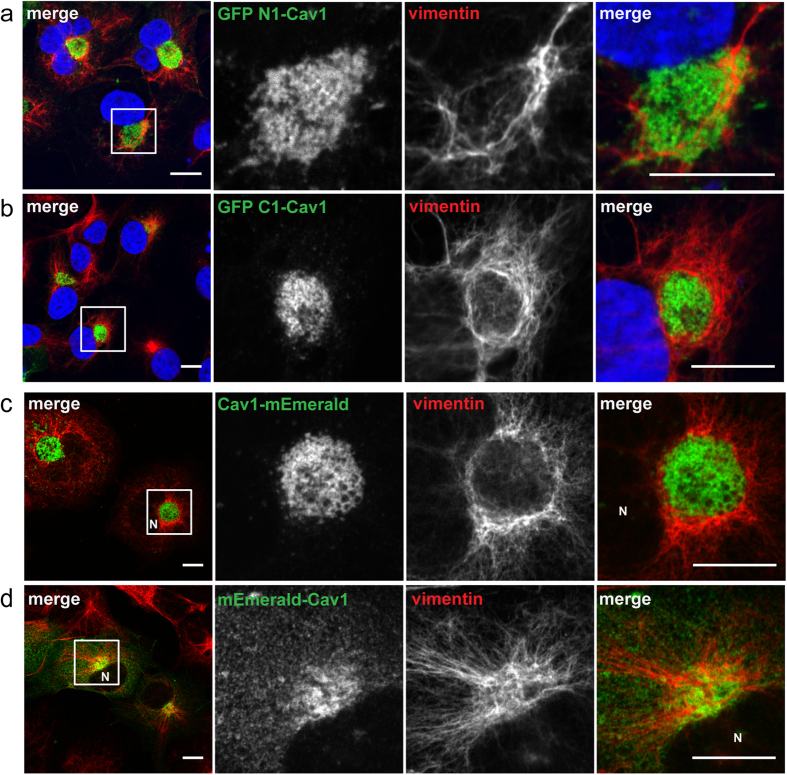
A variety of GFP-tagged Cav1 constructs induce aggresome formation. COS-7 cells were transiently transfected with the indicated Cav1 constructs (green) and stained with a mouse anti-vimentin antibody (red). Nuclei are either labeled in blue (**a,b**) or indicated by “N.” White boxes mark the areas of the zooms. Representative images are shown. Scale bars, 10 μm.

We found that several of these constructs also localized predominantly in perinuclear compartments that were surrounded by vimentin cages, suggesting that they too induce aggresome formation (Fig. 3, Supplementary Table S2). In particular, aggresome formation was observed for Cav1 constructs derived from both human and canine forms of Cav1, and tagged with either EGFP or mEmerald (Fig. 3a–c). However, not all of the constructs accumulated in aggresome (Fig. 3d). Interestingly, the presence of an EGFP tag at either the N or C-terminus of Cav1 induced aggresome formation (Fig. 3a,b), whereas mEmerald tagging induced aggresome formation only when located at C-terminus of Cav1 (Fig. 3c,d). Taken together, these results show that multiple Cav1 constructs can induce aggresome formation. They also indicate that the both the identity of the fluorescent protein tag and the tag orientation strongly influence whether Cav1 overexpression causes aggresome formation in COS-7 cells.

### Induction of aggresome formation by Cav1-GFP is cell type dependent

**Figure 4:**
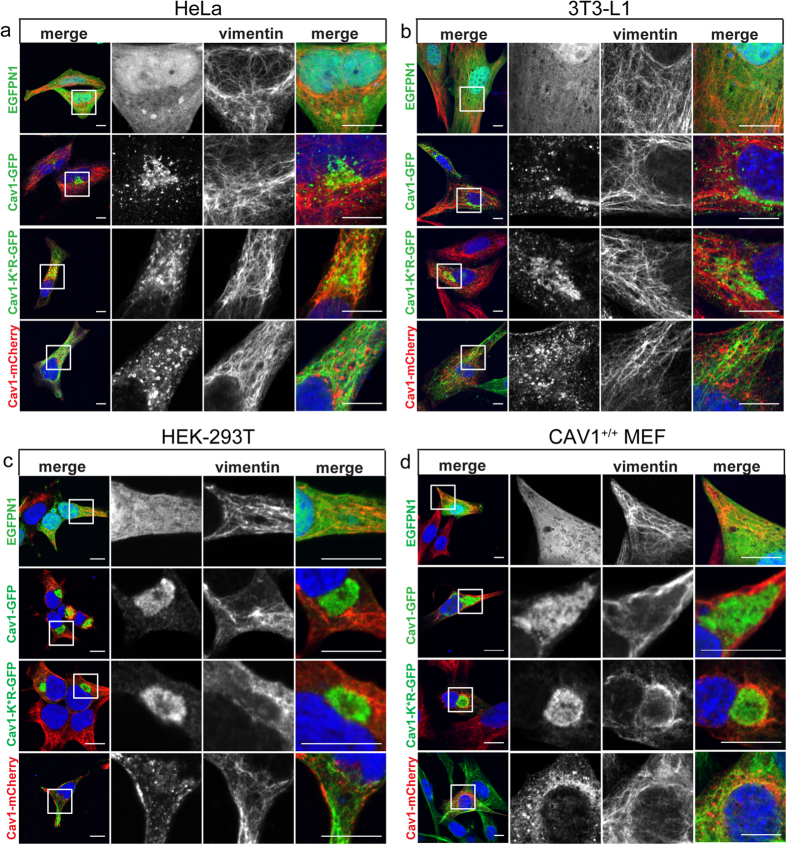
Cav1-GFP induces aggresome formation in a cell type-dependent manner. EGFPN1 or the indicated Cav1 constructs were transiently transfected into **(a)** HeLa, **(b)** 3T3-L1, **(c)** HEK-293T, or **(d)** Cav1^+/+^ MEFs. The cells were fixed and stained with either mouse anti-vimentin (HeLa, HEK-293T) or chicken anti-vimentin antibodies (3T3-L1 and Cav1^+/+^ MEFs). DRAQ5 was used mark the nucleus. In the merged images of cells expressing GFP constructs, GFP fluorescence is shown in green, vimentin is shown in red, and nuclei are labeled blue. For the Cav1-mCherry transfected cells, mCherry fluorescence is shown in red, vimentin staining is green, and nuclei are labeled blue. White boxes indicate the areas of the zooms. Representative images are shown. Scale bars, 10 μm.

We next tested whether aggresome formation is only observed in COS-7 cells or if this is a more general phenomenon that occurs in multiple cell types. For these studies we used the Cav1-GFP construct as a model, and Cav1-mCherry and EGFPN1 were used as controls. We found that overexpression of Cav1-GFP but not of EGFP or Cav1-mCherry drives aggresome formation in several commonly studied cell types (Fig. 4, Supplementary Fig. S3). In particular, Cav1-GFP accumulated in a perinuclear compartment that was surrounded by a vimentin cage in HEK-293T cells, Cav1^+/+^ MEFs, and Cav1^−/−^ MEFs (Fig. 4c,d, Supplementary Fig. S3, Supplementary Table S3). However, this was not the case in either HeLa or 3T3-L1 cells overexpressing Cav1-GFP (Fig. 4a,b, Supplementary Table 3). We conclude that the propensity of overexpressed Cav1-GFP to induce aggresome formation is cell type dependent.

### Ubiquitination of Cav1 is not required to target Cav1-GFP to aggresomes

Ubiquitinated proteins are often enriched in aggresomes, although ubiquitination is not an absolute requirement for accumulation of a given protein within aggresomes^19^,^23^,^29^. Thus, we next asked whether i) aggresomes induced by Cav1-GFP overexpression contain high levels of ubiquitinated proteins and ii) if ubiquitination of Cav1-GFP is required to target the protein to aggresomes.

**Figure 5:**
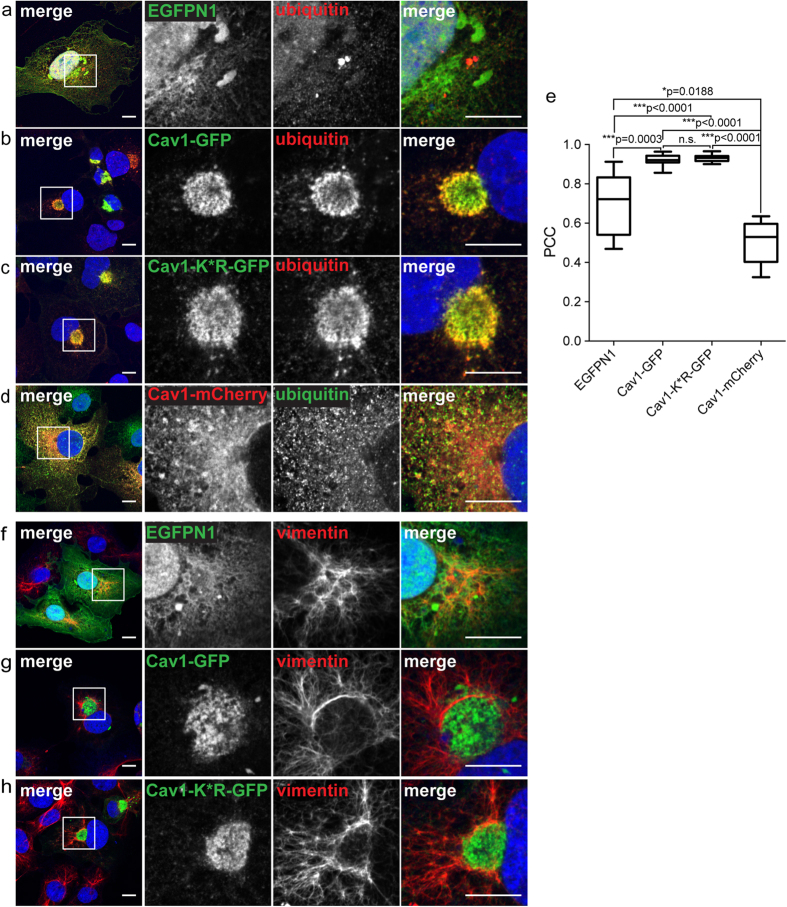
The perinuclear pool of Cav1-GFP is enriched in ubiquitinated proteins. COS-7 cells transiently transfected with empty vector (EGFPN1), Cav1-GFP, Cav1-K*R-GFP or Cav1-mCherry were stained with either an anti-ubiquitin antibody **(a–d)** or chicken anti-vimentin antibody **(f–h).** In the merged images, GFP fluorescence is shown in green and antibody labeling is shown in red. For the Cav1-mCherry transfected cells, mCherry fluorescence is shown in red and antibody staining in green. DRAQ5 staining was used to label the nucleus (blue). White boxes indicate the areas of the zooms. Scale bars, 10 μm. **(e)** Quantification of colocalization of ubiquitin with the indicated constructs using Pearson’s correlation coefficient (PCC). n.s., not significant, P > 0.05; *P ≤ 0.05; **P ≤ 0.01, ***P ≤ 0.001; Mann-Whitney U-test. n = 9 cells per condition. Note that EGFP shows a high degree of colocalization with ubiquitin because both proteins are distributed throughout the cytoplasm and nucleus.

To test for levels of ubiquitinated proteins associated with aggresomes, we performed immunostaining experiments. In COS-7 cells expressing Cav1-GFP, we observed strong labeling of perinuclear Cav1-GFP by endogenous ubiquitin, whereas ubiquitin was distributed throughout both the nucleus and cytoplasm of cells expressing EGFP (Fig. 5a,b,e). Thus, ubiquitinated proteins are indeed enriched in aggresomes induced by Cav1-GFP expression.

To determine whether ubiquitination of Cav1 itself is required for aggresome formation, we examined a lysine null Cav1 mutant that cannot be ubiquitinated, Cav1-K*R-GFP^17^. Like Cav1-GFP, the Cav1-K*R-GFP mutant accumulated in the perinuclear region of COS-7 cells and was surrounded by vimentin cages (Fig. 5g,h). A similar result was obtained in HEK-293T and Cav1^+/+^ MEFs (Fig. 4c,d). Cav1-K*R-GFP-positive aggresomes in COS-7 cells were also strongly labeled by ubiquitin (Fig. 5c,e). These findings suggest that ubiquitination of overexpressed Cav1 itself is not required to induce aggresome formation and that these aggresomes contain other ubiquitinated proteins.

Because Cav1 forms oligomers, it is possible that ubiquitination of endogenous Cav1 could potentially contribute to aggresome formation in response to overexpression of Cav1-K*R-GFP in COS-7 cells and other cell types in which endogenous Cav1 is present. To test this, we repeated this experiment in Cav1^−/−^ MEFs. The localization of Cav1-K*R-GFP was similar to that of Cav1-GFP and was also surrounded by a vimentin cage, suggesting it induces aggresome formation (Supplementary Fig. 3). This finding provides further support for a model in which ubiquitination of overexpressed Cav1 per se is not required to induce aggresome formation.

### Proteasomes and VCP accumulate in aggresomes induced by Cav1-GFP expression

**Figure 6:**
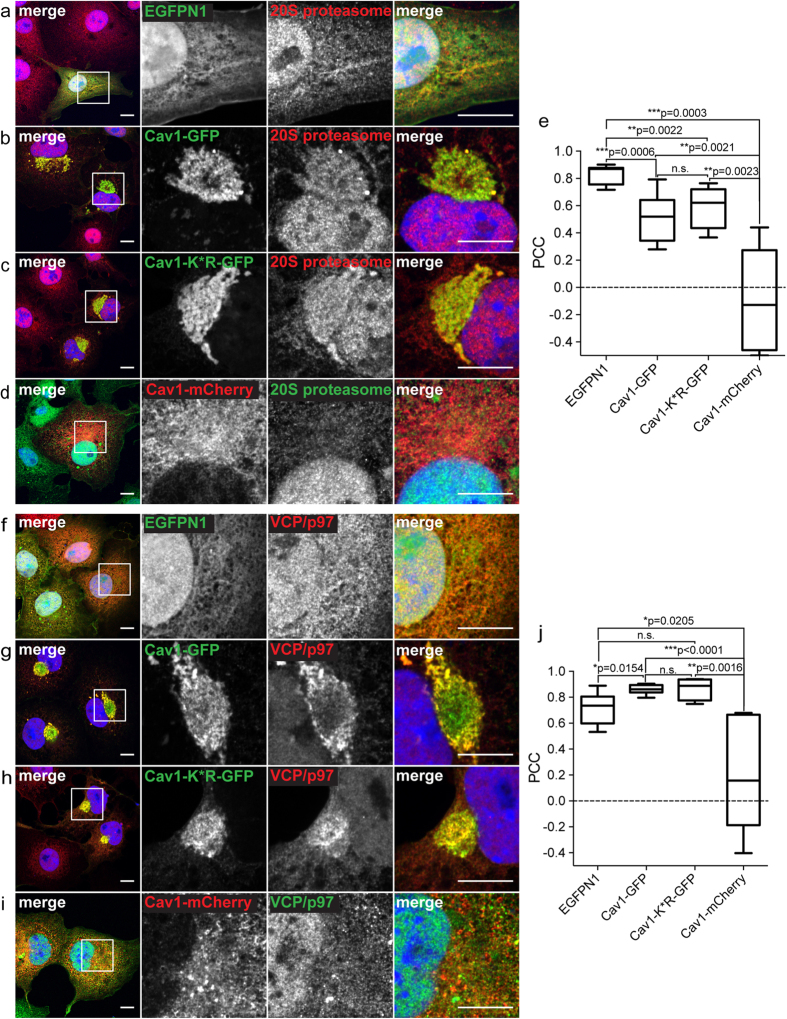
Proteasomes and VCP/p97 are recruited to aggresomes induced by overexpression of Cav1-GFP in COS-7 cells. COS-7 cells transiently transfected with empty vector (EGFPN1), Cav1-GFP, Cav1-K*R-GFP or Cav1-mCherry were stained with either anti-20S proteasome antibody **(a–d**), or anti-VCP/p97 antibody **(f–i).** GFP fluorescence is shown in green and antibody labeling is shown in red. For the Cav1-mCherry transfected cells, mCherry fluorescence is shown in red and antibody staining in green. DRAQ5 staining was used to label the nucleus (blue). White boxes indicate the areas of the zooms. Representative images from more than 4 independent experiments are shown. Scale bars, 10 μm. **(e,j)** Quantification of colocalization of 20S proteasome or VCP/p97 with the indicated constructs using Pearson’s correlation coefficient (PCC). n.s., not significant, P > 0.05; *P ≤ 0.05; **P ≤ 0.01, ***P ≤ 0.001; Mann-Whitney U-test. n = 5–11 cells per condition. Note that EGFP shows a high degree of colocalization with both 20S proteasomes and VCP/p97 because of their similar diffuse distribution in the cytoplasm and nucleus.

We next tested whether the aggresomes induced by Cav1-GFP overexpression contain other common aggresomal markers. Proteasomes often become concentrated at aggresomes and can play a role in clearance of aggresome-accumulated aggregates^29^. Consistent with this, we observed that a significant pool of 20S proteasomes was associated with Cav1-GFP-positive aggresomes in the perinuclear area of COS-7 cells (Fig. 6b,e). In contrast, 20S proteasomes were diffusely distributed in the nucleus and cytoplasm in cells overexpressing EGFPN1 (Fig. 6a) or Cav1-mCherry (Fig. 6d). 20S proteasome staining was also enriched in perinuclear aggresomes in COS-7 cells overexpressing Cav1-K*R-GFP (Fig. 6c,e) and in Cav1^−/−^ MEF cells expressing Cav1-GFP (Supplementary Fig. S3), but not in Cav1^−/−^ MEFs expressing the Cav1-GFP K*R mutant (Supplementary Fig. S3). Thus, 20S proteasomes are often, but not always associated with Cav1-GFP-induced aggresomes.

We also tested for the presence of valosin containing protein (VCP), also known as p97, in aggresomes. VCP plays an important role in protein degradation and aids in the delivery of ubiquitinated proteins to aggresomes^30^,^31^. VCP was uniformly distributed in the nucleus and cytoplasm of COS-7 cells expressing EGPN1 (Fig. 6f). In contrast, induction of aggresome formation by Cav1-GFP led to dramatic recruitment of VCP to the perinuclear region in COS-7 cells (Fig. 6g) as well as in COS-7 cells overexpressing Cav1-K*R-GFP (Fig. 6h). This was further confirmed by colocalization analysis (Fig. 6j). Thus, VCP is also a component of Cav1-GFP containing aggresomes.

### Aggresome-accumulated Cav1-GFP turns over rapidly

**Figure 7:**
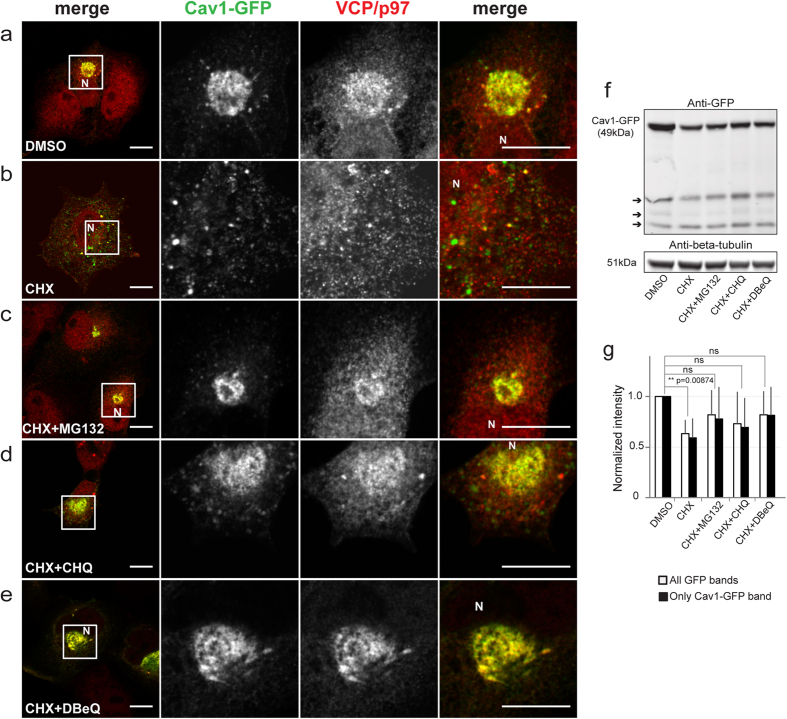
Cav1-GFP undergoes rapid turnover in aggresomes. COS-7 cells transfected with Cav1-GFP were immunostained with anti-VCP/p97 antibody after 6 h of treatment with **(a)** DMSO, **(b)** CHX (200 μg/ml), **(c)** CHX (200 μg/ml) + MG132 (10 μM), **(d)** CHX (200 μg/ml) + Chloroquine (CHQ) (100 μM), or **(e)** CHX (200 μg/ml) + DBeQ (10 μM). Images were acquired using similar microscope settings for DMSO, CHX and CHX+ inhibitor-treated cells. Representative images from 2 independent experiments are shown. In the merged images Cav1-GFP is shown in green and VCP/p97 immunostaining is shown in red. The position of the nucleus is marked with “N.” White boxes indicate the areas of the zooms. Scale bars, 10 μm. **(f)** Western blot of total cell lysates from COS-7 cells transfected with Cav1-GFP and treated with DMSO, CHX or CHX containing the indicated inhibitors. Blot was probed using an anti-GFP antibody. A representative blot is shown. Arrows point to putative Cav1-GFP degradation products. Control experiments verified that transfection efficiencies were similar for each sample (see methods for details.) **(g)** Quantification of normalized levels of Cav1-GFP from western blots from 3–4 independent experiments for each treatment.

Endogenous Cav1 has a half life ranging from 5 to 36 h^17^,^32^,^33^. We previously reported that the perinuclear pool of Cav1-GFP is rapidly turned over compared to endogenous Cav1 in caveolae in COS-7 cells^15^, suggesting that the recruitment of Cav1-GFP to this structure aids in its degradation. Aggresomally-targeted proteins can be degraded in several ways, including proteasomal and lysosomal-based mechanisms^29^,^30^,^34^,^35^. To determine the mechanism responsible for turnover of Cav1-GFP in aggresomes, we monitored GFP fluorescence by confocal microscopy following treatment of cells with cycloheximide (CHX) for 6 h to inhibit new protein synthesis (Fig. 7). Compared to DMSO (vehicle)-treated cells (Fig. 7a), CHX treatment led to a loss of both Cav1-GFP fluorescence and perinuclear VCP staining (Fig. 7b). To determine whether this clearance involves proteasome or lysosomal pathways, we treated cells with CHX, CHX plus MG132 to inhibit proteosomal degradation, or CHX plus chloroquine (CHQ) to inhibit lysosomal degradation. In addition, we also tested the requirement for VCP in this process by treating cells with a combination of CHX and DBeQ (N2, N4-dibenzylquinazoline-2,4-diamine), a VCP inhibitor^36^. In cells treated with MG132, CHQ, or DBeQ, Cav1-GFP fluorescence was partially restored within the perinuclear region (Fig. 7c–e). The residual pool of Cav1-GFP was also positive for VCP under these conditions (Fig. 7c–e). These results were verified independently by western blotting (Fig. 7f,g). Protein levels of Cav1-GFP were reduced in response to CHX treatment, whereas the addition of MG132, CHQ, or DBeQ protected Cav1-GFP from degradation (Fig. 7f,g). Together, these results imply that Cav1-GFP is cleared from the cells via a combination of mechanisms and that VCP assists in the clearance of the aggresomal pool of Cav1-GFP.

### Endogenous Cav1 is incorporated into aggresomes in response to proteotoxic stress

**Figure 8:**
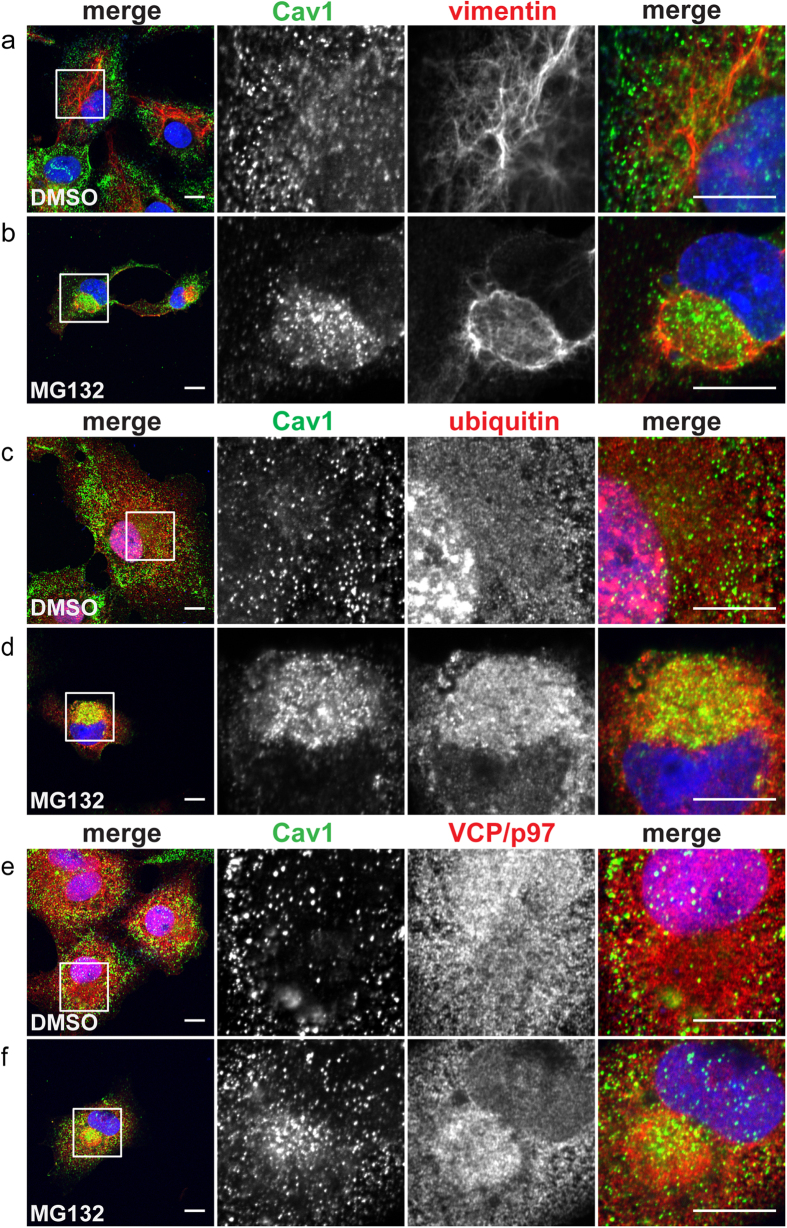
Endogenous Cav1 accumulates in aggresomes induced by proteotoxic stress. COS-7 cells treated with either DMSO **(a,c,e)** or 5 μM MG132 **(b,d,f)** for 24 h were immunostained with rabbit anti-Cav1 polyclonal antibody and vimentin (**a,b**), ubiquitin (**c,d**), or VCP/p97 (**e,f**). Images were acquired using similar microscope settings for DMSO- and MG132-treated cells. Representative images from 2 independent experiments are shown. In the merged images Cav1 staining is shown in green and vimentin, ubiquitin or VCP/97 staining is depicted in red. DRAQ5 staining was used to label the nucleus (blue). White boxes indicate the areas of the zooms. Scale bars, 10 μm.

Our results so far indicate that overexpressed Cav1-GFP accumulates within aggresomes where it is rapidly degraded. We wondered whether endogenous Cav1 is also prone to aggresome-mediated turnover. We tested this by treating untransfected COS-7 cells for 24 h with MG132, a proteosome inhibitor that has previously been shown to induce the formation of aggresomes^29^,^37^ (Fig. 8). As expected, vimentin cages formed in MG132 treated cells (Fig. 8b), and ubiquitin and VCP were also enriched within these aggresomes (Fig. 8d,f). Remarkably, in response to MG132 treatment endogenous Cav1 became enriched within the vimentin cages (Fig. 8b,d,f). Thus, endogenous Cav1 is also susceptible to aggresomal accumulation.

## Discussion

Multiple studies have shown that overexpression or mutations cause mistrafficking of Cav1, but to our knowledge the finding that the mistrafficked protein accumulates in aggresomes has never been previously reported. Here, we show that expression of Cav1-GFP leads to the perinuclear accumulation of the protein in a compartment with features characteristic of aggresomes. In particular, it is localized around the MTOC in a microtubule-dependent manner, surrounded by vimentin cages, and is enriched in ubiquitin, 20S proteasomes, and VCP/p97. We also find that targeting of Cav1-GFP to aggresomes does not require ubiquitination of overexpressed Cav1, since a lysine-less form of Cav1, Cav1 K*R-GFP, also induces aggresome formation in several cell types.

Our results suggest that the propensity of Cav1 to trigger aggresome formation appears to be highly dependent on how Cav1 is tagged. This is consistent with previous reports that tagging strategies can influence whether a protein will form aggresomes^21^. We found that Cav1-mCherry overexpression fails to generate aggresomes, whereas several different GFP-tagged Cav1 constructs derived from different species do (Supplementary Table S2). Why the fate of Cav1 is sensitive to whether it is tagged with variants of EGFP or mCherry remains to be determined. It is possible that this reflects differences in the ability of the fluorescent proteins to oligomerize^38^,^39^,^40^. Even though we found that the Cav1 construct tagged with a monomeric GFP variant, Cav1-mEmerald, has a similar localization as Cav1-GFP, some monomerized forms of GFP retain the ability to form oligomers when attached to oligomeric proteins^39^. We also observed differences in the behavior of some N- versus C-terminally tagged forms of Cav1. This could potentially impact whether the fluorescent proteins can interact with one another, or otherwise disrupt to folding or oligomerization of Cav1. In this regard, it is interesting to note that several studies have shown the C-terminus of Cav1 is important for oligomerization^41^,^42^ and mutations in the C-terminus of Cav1 are associated with human diseases^43^,^44^,^45^,^46^. Taken together, these findings suggest that the C-terminus of Cav1 is especially sensitive to manipulation.

We also found that aggresome induction in response to Cav1-GFP overexpression is cell type dependent. Aggresome formation was especially robust in COS-7 cells. However, Cav1-GFP also induced aggresome formation in several other cell types, including HEK-293T cells, Cav1^+/+^ MEFs, and Cav1^−/−^ MEFs (Supplementary Table S3). Interestingly, HeLa cells, which have been used in a number of studies of Cav1-GFP trafficking, did not show evidence for accumulation of Cav1-GFP within vimentin cages. This suggests that cell type-specific differences in the folding and/or trafficking mechanisms of Cav1, or differences in quality control machinery between cell types may ultimately determine whether overexpressed Cav1-GFP is targeted to aggresomes. The mechanistic basis for these cell type-specific differences remains to be discovered.

Our finding that Cav1-GFP overexpression induces aggresome formation has a number of important implications. First, aggresome induction by Cav1 constructs may be a widespread phenomenon. Inspection of published images reveals that intracellular accumulations of overexpressed Cav1 similar to those we report here are apparent in a number of studies^5^,^13^,^14^,^47^. We postulate that in many of these studies, mutant or overexpressed forms of Cav1-GFP were not actually enriched in the Golgi complex, but instead represent accumulation of the protein in aggresomes. Because it is easy to test for aggresome formation using markers such as vimentin, VCP, and ubiquitin, in future studies it should be straightforward to rule in or rule out whether they are induced under a given set of experimental conditions. Second, our findings raise the possibility that functional effects previously attributed to mutant or overexpressed forms of Cav1 overexpression could be the result of aggresome induction rather than mislocalization of Cav1 per se. For example, recruitment of components of the ubiquitin-proteosome pathway to aggresomes could potentially compete with their function in other pathways, and disrupt the overall proteostatic state of the cell. This could potentially help explain why it has been difficult to develop consensus models for the function of Cav1 and caveolae. Aggresome formation could also potentially be triggered when endogenous Cav1 is either overexpressed or mutated, a possibility that merits further study. This is especially important given the growing number of Cav1 mutations associated with human disease^43^,^44^,^45^,^46^. Finally, our results indicate that conditions that favor aggresome formation, such as proteasome inhibition, can lead to the trapping of endogenous wild type Cav1 in aggresomes. As such, it will be important to consider possible connections between Cav1 and other aggresome-inducing conditions in future studies.

## Materials and Methods

### Reagents

Cav1-GFP, Cav1-mCherry, and Cav1-myc were as described previously^15^,^16^. Details of different Cav1 constructs used are described in Supplementary Table S1. Rabbit anti-Cav1 polyclonal antibody (catalog number 610059) and mouse monoclonal (mAb) anti-Cav1 clone 2234 (mAb 2234, catalog number 610494) were purchased from BD Biosciences. Mouse anti-Myc (9B11) was procured from Cell Signaling. Rabbit Anti-Giantin (# ab24586) was purchased from AbCam, whereas mouse anti-Golgin97 (Clone CDF4) was obtained from Thermo Scientific. Mouse anti-Vimentin (# V6389) was purchased from Sigma. Chicken anti-vimentin (Clone Poly 29191) was purchased from Biolegend. Mouse anti-Ubiquitin (Fk2) (# BML-PW8810-0500) and anti-20S proteasome antibodies (# BML-PW8115-0025) were procured from Enzo Life Sciences. Mouse anti-VCP/p97 (# NB120-11433) antibody was purchased from Novus Biological. Anti-GFP antibody (Clone JL8) was purchased from Geneclone. Anti-Beta tubulin antibody E7 was procured from DSHB, UIOWA. MG132, Nocodazole, DMSO and chloroquine were purchased from Sigma. Cycloheximide was procured from MP Biomedicals. VCP/p97 inhibitor DBeQ was procured from Tocris. Lipofectamine 2000 and Prolong Gold anti-fade mounting reagent were obtained from Life Technologies.

### Cell culture and transfections

COS-7, HeLa, wild type (WT) MEFs and Caveolin-1 null (Cav1^−/−^) MEFs were obtained from ATCC and cultured in DMEM supplemented with 10% fetal bovine serum (FBS), 1% Pen/Strep at 37 °C and 5% CO_2_ in a tissue culture incubator. DMEM, FBS, 0.05% Trypsin and Pen/strep were purchased from Gibco (Life Technologies). 3T3-L1 cells were cultured in DMEM containing 10% bovine calf serum, 1% HEPES and 1% Pen/Strep.

Cells were plated one day prior to transfection. Transient transfections were performed using Lipofectamine 2000 as per the manufacturer’s instructions. One microgram of DNA was used for individual wells of six-well plates and 2 μg was used for 6 cm dishes. Unless otherwise indicated, cells were transfected 1 day prior to experiments.

### Immunofluorescence microscopy

Cells grown on glass coverslips were processed for immunofluorescence after 24 hours of transfection with different plasmids. Briefly, the cells were fixed at 37 °C for 15 min in 4% PFA in PBS. Post fixation, the cells were quenched by three rinses in 100 mM glycine in PBS. The cells were then permeabilized and blocked in blocking buffer composed of 0.1% TX-100 in PBS containing 5% glycine and 5% normal goat or donkey serum for 60 min at RT. For staining with chicken anti-vimentin antibody the cells were permeabilized with 0.1% saponin as suggested by manufacturer. Unless otherwise indicated cells were incubated with indicated primary antibodies at a dilution of 1:100 in blocking buffer with 0.05% Tx-100 for 1–2 h at RT. After rinsing in PBS coverslips were incubated for 1 h in a 1:300 dilution of Alexa Fluor 488-, Alexa Fluor 546-, and Alexa Fluor 647-conjugated secondary antibodies as required. Where indicated, the cells were incubated with nuclear stain DRAQ5 to label the nucleus. After washing in PBS the coverslips were mounted on glass slides using Prolong Gold antifade reagent. Fluorescent images were acquired using a Zeiss LSM 510 confocal microscope (Carl Zeiss Microscopy, Inc.; Thornwood, NY) using either a Plan-Apochromat 100X/1.4 oil or a 40 X/1.3 NA Zeiss Plan-Neofluar oil immersion objective, an Argon/2 30 mW laser (458, 488, 514 nm) or HeNe lasers (543 nm or 633 nm), and filter sets provided by the manufacturer. For presentation purposes, images were processed using ImageJ software.

### Line profile analysis

Where indicated, image intensity profiles were generated using the “Plot Profile” command of ImageJ. The intensity values of each pixel was normalized as


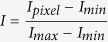


where I_max_ and I_min_ are the maximum and minimum intensity values along the line and I_pixel_ is the intensity of each pixel.

### Colocalization Analysis

Colocalization analysis was performed by calculating Pearson’s Correlation coefficients for 4X zoom images using the Intensity Correlation Analysis plugin^48^ in Fiji^49^. For each condition, 5–11 images were analyzed from two independent experiments, unless stated otherwise in the figure legends. Pearson’s Correlation Coefficients are reported as Box and Whisker plots. Using Prism 6 GraphPad software, a two-tailed, unpaired, non-parametric Mann-Whitney U-test was used to calculate p-values.

### Stochastic Optical Reconstruction Microscopy (STORM)

For STORM based imaging, COS-7 cells grown in MatTek dishes were transfected with the indicated plasmids. 24 h post transfection the cells were fixed with 4% PFA containing 0.1% glutaraldehyde. Following blocking and permeabilization with 1% BSA and 0.1% Triton-X-100 for 1 h, the cells were labeled with anti-giantin rabbit antibody and subsequently with Alexa 647 conjugated goat-anti rabbit secondary antibody as described for the immunofluorescence imaging section. Super resolution imaging was performed in a reducing buffer (50 mM Tris, 10 mM NaCl, 10% glucose, 500 μg/mL glucose-oxidase, 40 μg/mL catalase, and 1% β- mercaptoethanol at pH 8.5). Cells were imaged using an inverted Nikon N microscope equipped with a Perfect Focus System to reduce drift and a 100x Apo TIRF 1.49/NA oil objective. A dual colored epifluorescence image of the cell was collected using low laser power under Hilo conditions prior to the STORM imaging. For STORM images, a ROI corresponding to the Golgi complex was excited using a 640 nm laser excitation and imaged using Andor iXon Ultra 897 EMCCD camera. To image the cytosolic region of the cell, off-TIRF imaging was performed to ensure greater penetration depth for imaging close to the nucleus. For image reconstruction, at least 10000 frames per cell were acquired. All experiments were performed twice with a sample size of at least 5 cells imaged and analyzed for each trial.

### Stochastic Optical Reconstruction Microscopy Analysis

Analysis of the images were performed using ThunderSTORM plugin^50^ in FIJI-ImageJ^49^,^51^. The images were first filtered using a B-Spline Wavelet Filter (order 3 and scale 2). We used Local Maxima to determine the approximate localization of molecules and an Integrated Gaussian Point Spread Function for the accurate localization of the emitters. After localization, the localizations that fell within the localization error were removed. The emitters that were localized within 20 nm within 1 off frame were merged. Finally, drift was corrected using cross correlation.

### Drug treatments

Cycloheximide and inhibitor treatments were carried out 24 hours after transfection. Transfected cells were incubated at 37 °C with 200 μg/mL cycloheximide in DMSO for 6 h. For some experiments, cells were treated with cycloheximide (200 μg/mL in DMSO) containing either the proteasomal inhibitor MG132 (10 μM in DMSO), lysosomal inhibitor choloroquine (CHQ) (100 μM in water), or the VCP/p97 inhibitor DBeQ (10 μM in DMSO) for 6 h at 37 °C prior to fixation and processing for immunofluorescence. To induce aggresome formation by proteosomal inhibition, untransfected COS-7 cells plated on glass coverslips were incubated with MG132 (5 μM in DMSO) for 24 h prior to fixation and processing for immunofluorescence. As controls for the drug treatments, cells were treated with equivalent amount of DMSO (vehicle). For nocodazole treatment, the transfected cells were incubated with 5 μg/ml nocodazole for 15 min at 4 °C and then shifted to 37 °C for 1 h prior to fixation and processing for immunofluorescence labeling and imaging.

### Western blotting

COS-7 cells transfected with the indicated Cav1 constructs were treated with DMSO or inhibitors as described above and the cells were scraped and lysed in RIPA buffer (Sigma Aldrich) with cocktail protease inhibitor (Roche) at 4 °C. The debris was spun down at 13,000 rpm for 20 min. After total protein quantification using BCA reagent (Thermo Scientific), equal amounts of DMSO-treated and inhibitor-treated protein lysates were processed for western blotting. Electrophoretic transfer was conducted using Novex^®^ NuPAGE^®^ SDS-PAGE Gel System (Life Technologies). NuPAGE^®^ 4–12% gels (Life Technologies) were used for the protein separation. SeeBlue^®^ Pre-stained Protein (Life Technologies) was used as molecular weight standards. After blocking in blocking buffer, the PVDF membranes (from Millipore) were probed with the indicated antibodies and a LI-COR Odyssey infrared imaging system (LI-COR Biosciences) was used to visualize the blots. Quantification of Western blot images was performed using ImageJ.

### Quantification of transfection efficiencies

For calculating the transfection efficiencies for western blot experiments a 12 mm coverslip was placed in 6 cm plates marked for treatments with different inhibitors. Equal number of cells were seeded in each plate and transfected with Cav1-GFP plasmid. 24 hours post transfection the coverslips from individual plates were transferring into 24 well plates and fixed with 4%PFA. The cells in the 6 cm plates were then treated with different inhibitors as described above. Post fixation the coverslips were blocked with blocking solution as described for immunofluorescence experiments and stained with DRAQ5 to label nucleus. The coverslips were the mounted on glass slides and imaged. Fluorescent images were acquired using a Zeiss LSM 510 confocal microscope (Carl Zeiss Microscopy, Inc.; Thornwood, NY) using a Plan-Apochromat a 40 X/1.3 NA Zeiss Plan-Neofluar oil immersion objective. Images were acquired for 10 randomly selected fields of view for a given coverslip. The number of cells per field was quantified using the Cell Counter plugin of Image J and the cells were manually scored as being positive or negative for Cav1-GFP.

## Additional Information

**How to cite this article**: Tiwari, A. *et al*. Caveolin-1 is an aggresome-inducing protein. *Sci. Rep.* **6**, 38681; doi: 10.1038/srep38681 (2016).

**Publisher's note:** Springer Nature remains neutral with regard to jurisdictional claims in published maps and institutional affiliations.

## Supplementary Material

Supplementary Material
