# Supplementary material for: Caveolin-1 is an aggresome-inducing protein
Source: Sci Rep. 2016 Dec 8;6:38681. doi: 10.1038/srep38681 (PMC5144149; doi:10.1038/srep38681)
Supplement: Supplementary Material [file srep38681-s1.pdf]

Supplementary Information

Ajit Tiwari, Courtney A. Copeland, Bing Han, Caroline A. Hanson, Krishnan Raghunathan, and Anne K. Kenworthy

"Caveolin-1 is an aggresome-inducing protein"

Supplementary Table S1: Caveolin-1 (Cav1) constructs used in this study

Construct	Vector Backbone	Promoter	Gene Bank ID of Insert	Species of CAV1-cDNA insert	Addgene Catalogue Number	Reference
Cav1-myc	pCDNA 3.1	CMV	NM_001753	*Homo sapiens*	--	1
Cav1-GFP	pEGFP-N1 (Clontech)	CMV	NM_001003296	*Canis familalis*	--	2
Cav1-mCherry	pmCherry-N1 (Clontech)	CMV	NM_001003296	*Canis familalis*	--	3
Cav1-K*R-GFP (Cav1 lysine null mutant)	pmEGFP-N1	CMV	NM_001753	*Homo sapiens*	#27766	4
GFP N1-Cav1	pEGFP-N1 (Clontech)	CMV	NM_001003296	*Canis familalis*	--	5
GFP C1-Cav1	pEGFP-C1 (Clontech)	CMV	NM_001003296	*Canis familalis*	--	5
Cav1-mEmerald	N1 Cloning vector (Clontech)	CMV	NM_001753	*Homo sapiens*	#54026	3
mEmerald-Cav1	C1 Cloning vector (Clontech)	CMV	NM_001753	*Homo sapiens*	#54025	3

1 Machleidt, T., Li, W. P., Liu, P. & Anderson, R. G. Multiple domains in caveolin-1 control its intracellular traffic. *J. Cell Biol.* 148, 17-28, (2000).

2 Pelkmans, L., Kartenbeck, J. & Helenius, A. Caveolar endocytosis of simian virus 40 reveals a new two-step vesicular-transport pathway to the ER. *Nat Cell Biol* 3, 473-483, (2001).

3 Hanson, C. A. *et al.* Overexpression of caveolin-1 is sufficient to phenocopy the behavior of a disease-associated mutant. *Traffic* 14, 663-677, (2013).

4 Hayer, A., Stoeber, M., Bissig, C. & Helenius, A. Biogenesis of caveolae: stepwise assembly of large caveolin and cavin complexes. *Traffic* 11, 361-382, (2010).

5 Volonte, D., Galbiati, F. & Lisanti, M. P. Visualization of caveolin-1, a caveolar marker protein, in living cells using green fluorescent protein (GFP) chimeras. The subcellular distribution of caveolin-1 is modulated by cell-cell contact. *FEBS Lett* 445, 431-439, (1999).

Supplementary Table S2: Comparison of aggresome induction by different Cav1 constructs in COS-7 cells based on the presence (+) or absence (-) of vimentin cages.

Cav1-Construct	Species	Vimentin Cage	Figure
Cav1-GFP	*Canis familalis*	+	Figure 2b, 5g
Cav1-K*R-GFP	*Homo sapiens*	+	Figure 5h
GFP N1-Cav1	*Canis familalis*	+	Figure 3a
GFP C1-Cav1	*Canis familalis*	+	Figure 3b
Cav1-mEmerald	*Homo sapiens*	+	Figure 3c
mEmerald-Cav1	*Homo sapiens*	-	Figure 3d

Supplementary Table S3: Cell type dependence of aggresome induction by Cav1-GFP or Cav1-K*R-GFP as assayed by the presence (+) or absence (-) of vimentin cages

Cell Type	Cav1-Construct	Vimentin Cage	Figure
COS-7	Cav1-GFP	+	Figure 2b, 5g
	Cav1-K*R-GFP	+	Figure 5h
HeLa	Cav1-GFP	-	Figure 4a
	Cav1-K*R-GFP	-	Figure 4a
$Cav1^{+/+}$ MEF	Cav1-GFP	+	Figure 4d
	Cav1-K*R-GFP	+	Figure 4d
$Cav1^{-/-}$ MEF	Cav1-GFP	+	Supplementary Figure S3
	Cav1-K*R-GFP	+	Supplementary Figure S3
HEK 293T	Cav1-GFP	+	Figure 4c
	Cav1-K*R-GFP	+	Figure 4c
3T3-L1	Cav1-GFP	-	Figure 4b
	Cav1-K*R-GFP	-	Figure 4b

Supplementary Figure S1

a

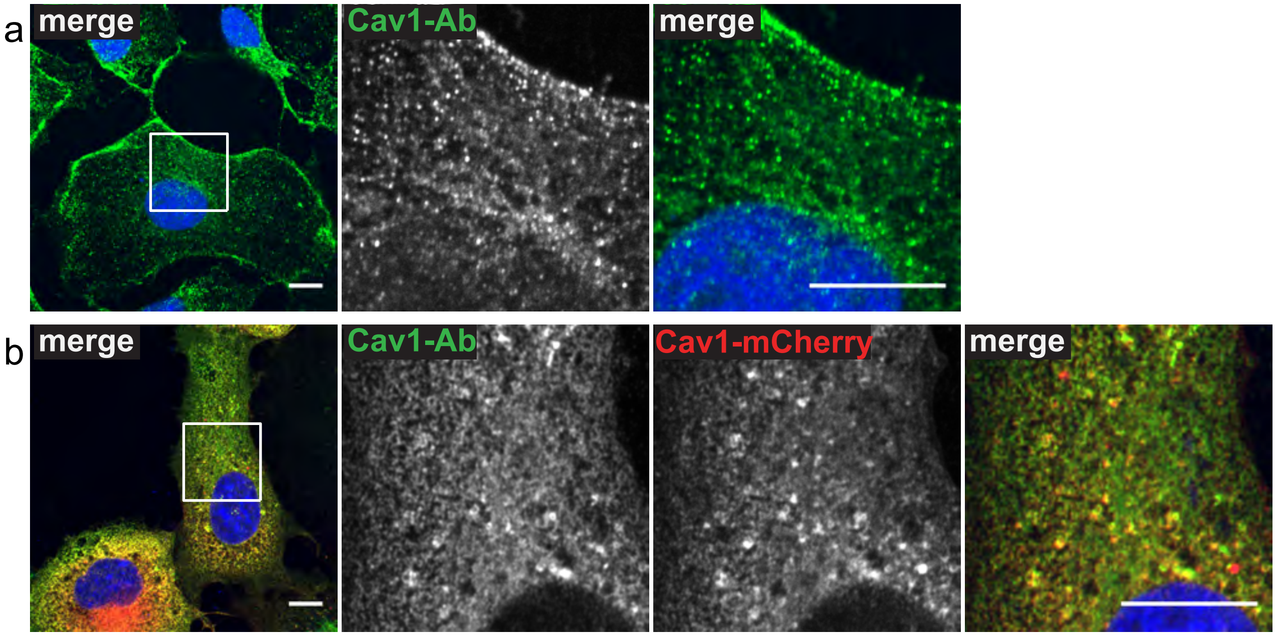

Supplementary Figure 1: Antibody staining of Cav1 in COS-7 cells. COS-7 cells were either left untransfected **(a)** or transfected with Cav1-mCherry **(b)**. The cells were fixed and stained using an anti-Cav1 rabbit polyclonal antibody. In the merged images, Cav1 antibody staining is shown in green, Cav1-mCherry fluorescence is shown in red, and the nucleus is labeled blue. White boxes indicate the areas of the zooms. Scale bars, 10 µm.

Supplementary Figure S2

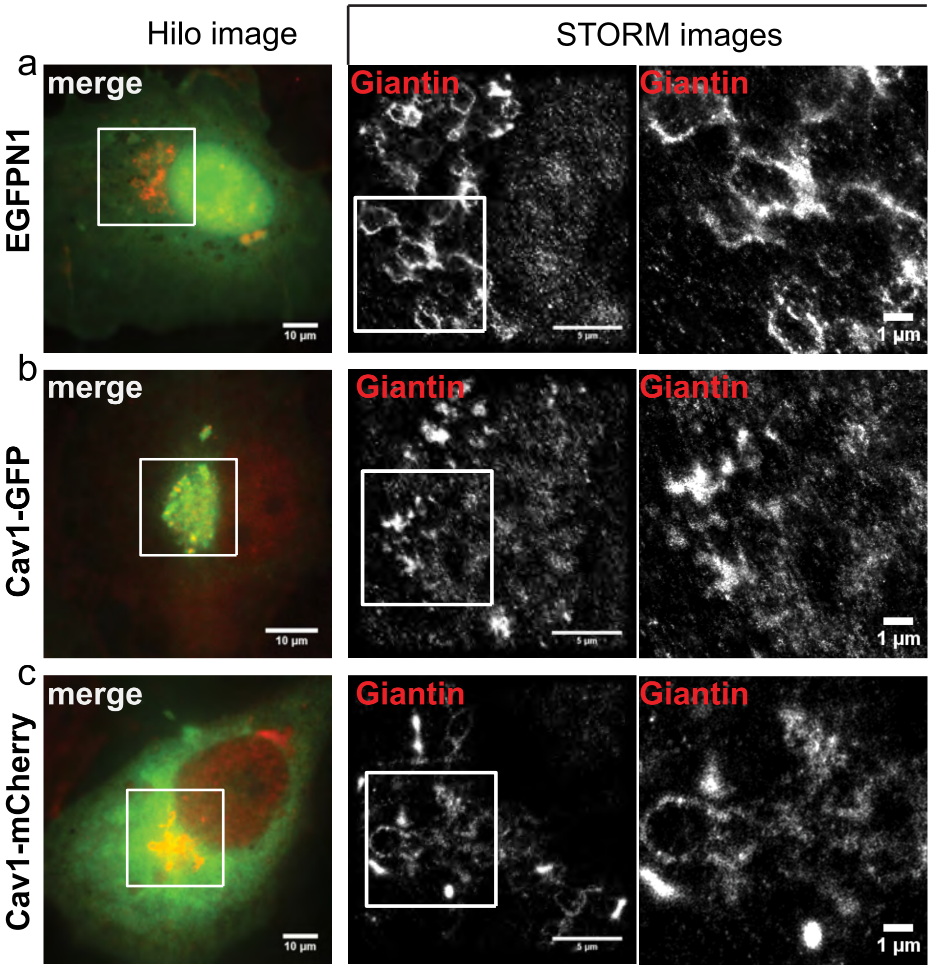

Supplementary Figure 2: Overexpression of Cav1-GFP disrupts Golgi structure. COS-7 cells transfected with the indicated constructs were fixed and stained with an anti-giantin antibody. Cells were first imaged with low laser power under Hilo conditions for the representative images and then subsequently imaged under high laser power to enable photoconversion for super-resolution images. The images were then processed using standard STORM imaging protocols as described in the methods section. Images show representative cells expressing **(a)** EGFPN1, **(b)** Cav1-GFP, or **(c)** Cav1-mCherry in green and giantin immunostaining in red. Hilo images are shown in the left panels. Reconstructed STORM images showing the Golgi morphology of the boxed regions are shown in the middle panels. The panels on the right show an enlarged image corresponding to the inset from the STORM image. Images are representative of two independent experiments. Scale bars for the Hilo images are 10µm. For the reconstructed STORM image and its corresponding enlarged inset, the scale bars are 5µm and 1µm, respectively.

Supplementary Figure S3

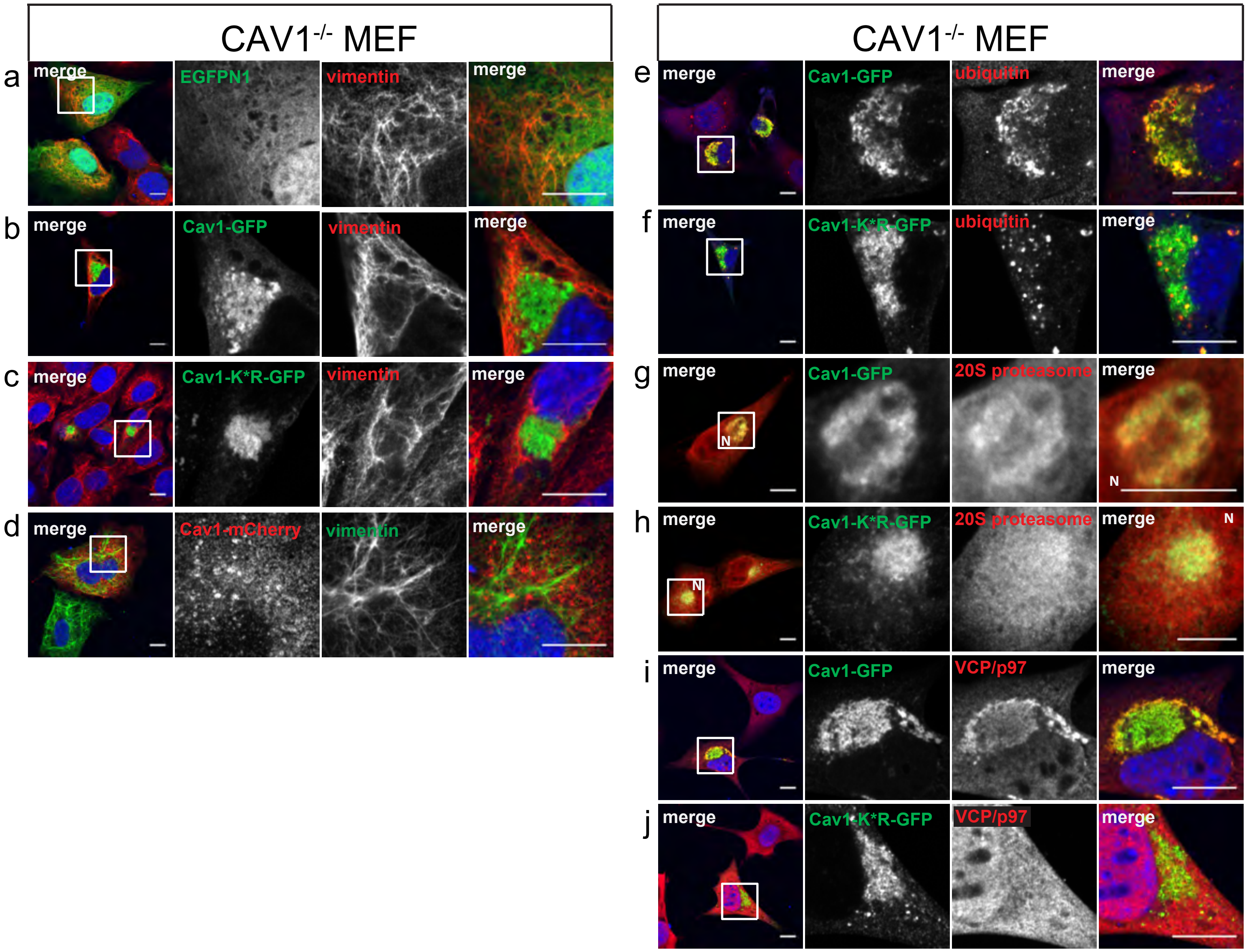

Supplementary Figure 3: Overexpression of Cav1-GFP induces the perinuclear accumulation of aggresomal markers in cells lacking endogenous Cav1. Cav1$^{-/-}$ MEFs transfected with the indicated Cav1 constructs were fixed and stained for **(a-d)** vimentin, **(e, f)** ubiquitin, **(g, h)** 20S proteasomes, or **(i, j)** VCP/p97. In the merged images, Cav1-GFP and Cav1 K*R-GFP are shown in green, Cav1-mCherry is shown in red, antibody staining is depicted in red or green as indicated on the figure, and nuclei are labeled blue. White boxes indicate the areas of the zooms. Representative images from 2 independent experiments are shown. Scale bars, 10 µm. Note that unlike Cav1-GFP, Cav1-K*R-GFP-containing aggresomes were not strongly labeled with ubiquitin, 20S proteasomes, or VCP/p97. This implies that ubiquitination of Cav1-GFP itself is required for other aggresomal markers proteins to become enriched in aggresomes in an otherwise Cav1 null background.
